# Decoding in the Fourth Dimension: Classification of Temporal Patterns and Their Generalization Across Locations

**DOI:** 10.1002/hbm.70152

**Published:** 2025-01-30

**Authors:** Alejandro Santos‐Mayo, Faith Gilbert, Laura Ahumada, Caitlin Traiser, Hannah Engle, Christian Panitz, Mingzhou Ding, Andreas Keil

**Affiliations:** ^1^ Department of Psychology University of Florida Gainesville Florida USA; ^2^ Department of Psychology University of Bremen Bremen Germany; ^3^ J. Crayton Pruitt Family Department of Biomedical Engineering University of Florida Gainesville Florida USA

**Keywords:** decoding, multi‐variate pattern analysis, temporal patterns

## Abstract

Neuroimaging research has increasingly used decoding techniques, in which multivariate statistical methods identify patterns in neural data that allow the classification of experimental conditions or participant groups. Typically, the features used for decoding are spatial in nature, including voxel patterns and electrode locations. However, the strength of many neurophysiological recording techniques such as electroencephalography or magnetoencephalography is in their rich temporal, rather than spatial, content. The present report introduces the time‐GAL toolbox, which implements a decoding method based on time information in electrophysiological recordings. The toolbox first quantifies the decodable information contained in neural time series. This information is then used in a subsequent step, generalization across location (GAL), which characterizes the relationship between sensor locations based on their ability to cross‐decode. Two datasets are used to demonstrate the usage of the toolbox, involving (1) event‐related potentials in response to affective pictures and (2) steady‐state visual evoked potentials in response to aversively conditioned grating stimuli. In both cases, experimental conditions were successfully decoded based on the temporal features contained in the neural time series. Spatial cross‐decoding occurred in regions known to be involved in visual and affective processing. We conclude that the approach implemented in the time‐GAL toolbox holds promise for analyzing neural time series from a wide range of paradigms and measurement domains providing an assumption‐free method to quantifying differences in temporal patterns of neural information processing and whether these patterns are shared across sensor locations.

## Graphical abstract

Time‐GAL toolbox is a new decoding‐based toolbox for analyzing neurobiological time series such as EEG or MEG recordings. Its methodology uses the temporal information contained in neurophysiological activity to describe the spatial dependency along the brain locations during the trial time.

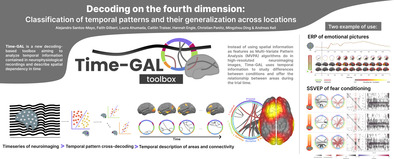

## 1 Introduction

The development of human neuroimaging tools such as functional magnetic resonance imaging (fMRI) and magneto‐ and electroencephalography (M/EEG) has transformed the study of human brain function. Traditionally, these measurements have been analyzed using univariate statistics (Friston, Jezzard, and Turner 1994), in which a linear statistical model is applied to a given time point, voxel, or electrode. Because of the univariate nature of this approach, it disregards information regarding spatial or temporal patterns (Mahmoudi et al. 2012). Thus, multivariate pattern analysis (MVPA) has emerged as an alternative tool for discriminating the neural activity patterns related to a given stimulus, experimental condition, or participant (Carrasco et al. 2024; Haxby 2012). The present toolbox paper introduces the conceptual background, goals, and implementation of the time‐GAL toolbox, a suite of functions written in Matlab, for multivariate pattern analysis of temporal features present in electrophysiological time series.

**FIGURE 1:**
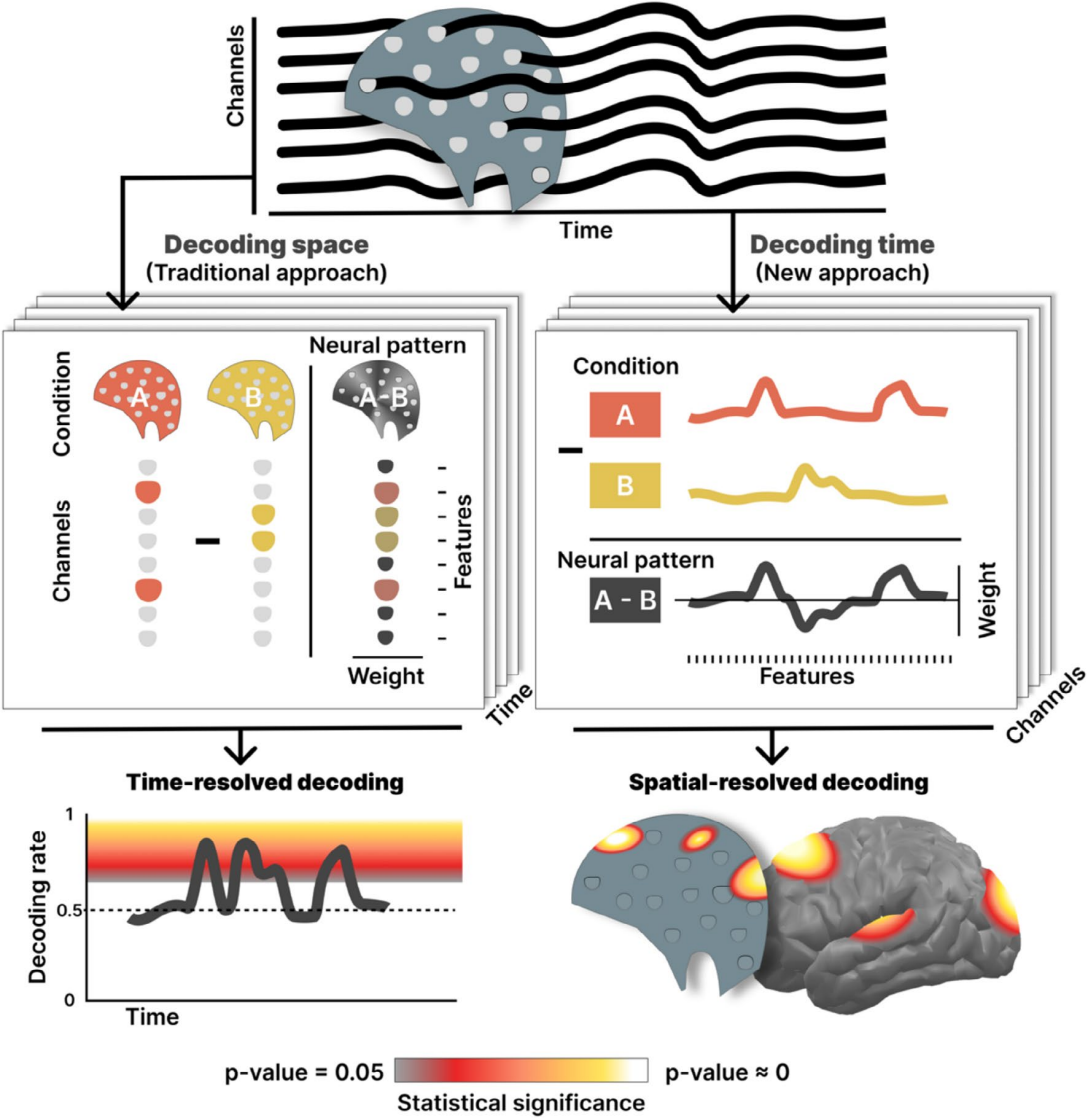
Flowchart describing how MVPA‐based decoding techniques may be carried out following the traditional approach based on spatial features (left) or the time‐GAL approach proposed here (right). Data from electrophysiological recordings (top) offer both spatial and temporal information in the form of channels and time series, respectively, which can be used for decoding. Thus, each procedure (middle panels) leverages one of the dimensions using its data to fit a classifier, repeating this operation over the other dimension to examine generalization. Specifically, the traditional approach employs channels or sensors as features; the time‐GAL approach uses temporal features. Finally, the results (bottom) for both the traditional (space‐based) and the time‐GAL method show the decoding performance across time or locations, respectively.

Multivariate decoding techniques capitalize on the information found in neural patterns to characterize the multifaceted properties that define a given brain process (Peelen and Downing 2023). Also known as decoding methods, these techniques were originally introduced for fMRI data (Haxby 2012; Haxby et al. 2001), and their use has steadily increased in recent years, becoming a standard procedure in this field (Peelen and Downing 2023; Rivolta et al. 2014). Meanwhile, in the field of M/EEG research, decoding approaches are also increasingly used (Grootswagers, Wardle, and Carlson 2017) with several standard procedures emerging (Ashton et al. 2022; Bae and Luck 2019; Bo et al. 2022). Typically, these standard procedures use spatial information to decode the underlying neural activity patterns (see Bode et al. 2019 for an exception), paralleling approaches used with fMRI data (see Figure 1 left). Other approaches use temporal and spatial features jointly, leveraging the potential of deep learning algorithms and machine learning approaches (Chard et al. 2024; Csaky et al. 2023). However, the resolution of spatial information is typically reduced for electrophysiological tools like EEG in comparison with fMRI. It is widely accepted that the primary strength of EEG and MEG recordings lies in their rich temporal information, rather than in their spatial distribution. Specifically, human electrophysiology reflects the time series of neural population activity arising from post‐synaptic events, subject to volume conduction and other distortions when measured from the scalp. The resulting waveforms contain information about the neural response and/or oscillatory rhythms related to a specific experimental condition or stimulus (David, Kilner, and Friston 2006).

In the present paper, we introduce the usage of the time‐GAL toolbox, applying MVPA algorithms to the temporal dimension of EEG signals, and thus taking advantage of the high resolution of neural time series data in this dimension. In this approach, we use all time points of each trial time series as features that enter a multivariate classifier model (see Figure 1, right), in contrast to spatial information (recording sites) which has largely been used in the literature previously. Then, we repeat this procedure for each recording location (e.g., electrodes in the case of EEG) to obtain a spatially resolved depiction of scalp regions in which the temporal dynamics related to a condition or stimulus contain overlapping decodable information based on temporal features. In a second step, we adapt a method proposed by King and Dehaene (2014) called generalization across time (GAT), in which a classifier algorithm is trained using spatial information for one (training) time point and then tested across a range of different time points. This procedure allows researchers to characterize the temporal stability of neural patterns related to specific conditions or stimuli. The time‐GAL approach, using the temporal waveform as a feature vector for classification allows us to quantify the extent to which different recording sites share decodable information. This is implemented as a generalization across location (GAL), in which a temporal decoder trained at one location is applied to other locations. By mapping the cross‐decoding accuracy across locations, the time‐GAL method offers a multivariate approach to defining the unique topographic patterns in which temporal dynamics are related to a specific stimulus or experimental condition. Finally, another important contribution of the current work consists of the combination of not only spatial, but also temporal information to the decoding output. Classifier models are considered backward models as they extract the information about experimental manipulations from the brain's responses to these manipulations. On the other hand, forward models describe the process in which experimental manipulations and brain biophysics lead to the recorded neurophysiological data. The exact weights of a classifier‐based backward model can only be extracted using a corresponding linear forward model (Haufe et al. 2014). Therefore, we implemented a simple linear forward model based on correlations of the data with the labels to visualize the dynamics of time‐GAL decoding and cross‐decoding patterns.

In the present report, we illustrate the usage of the time‐GAL toolbox, for analyzing neural time series data, using two EEG experiments. The experiments involved (1) affective picture viewing and (2) aversive generalization conditioning.

## 2 Methods

The time‐GAL toolbox was written in the MATLAB environment, a programming language widely used by researchers in the field of human neurosciences. Time‐GAL also uses an input format that is readily compatible with outputs provided by widely used MATLAB toolboxes for EEG and MEG preprocessing such as eeglab, erplab, or fieldtrip.

**FIGURE 2:**
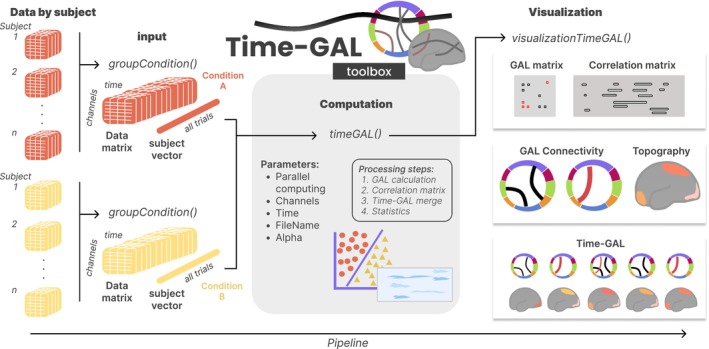
Flowchart describing the time‐GAL toolbox pipeline for preparing the time series data (left), for decoding the experimental conditions of interest (middle), and for visualizing the results (right). At the beginning of the process, data from the participants are loaded and grouped into a single three‐dimensional array with an accompanying vector indexing participants for each condition. These arrays serve as input for the timeGAL function. Finally, the output can be visualized, showing cross‐decoding matrices as well asstatic or temporally resolved connectivity and topography.

The time‐GAL toolbox represents a simple and easy‐to‐use implementation that allows users to arrange their data in a simple input array with three dimensions (sensors × time × trials). The main steps are implemented in a function called “timeGAL.m” in which the neural data matrices are input along with a list of labels indicating the mapping of trials onto participants and conditions (see Figure 2). In addition, functions are provided for calculating the forward model through Pearson's correlation and for combining its temporal information with GAL outputs.

The toolbox can be retrieved from https://github.com/csea‐lab/time‐GAL and example data used in this report may be downloaded from https://osf.io/q56ns. MATLAB live scripts for each example dataset (see below) provides a walk‐through for a typical analysis in which two conditions are being compared. Also, a more detailed documentation of the toolbox can be consulted in https://osf.io/q56ns/wiki or https://github.com/csea‐lab/time‐GAL/wiki. In the following sections, we describe the methods and implementation of time‐GAL.

### 2.1 Example Datasets

To provide a demonstration of the time‐GAL procedure, two datasets were used, involving two types of electrophysiological response: event related potentials (ERP) and steady‐state visual evoked potentials (ssVEP). Both datasets are available at https://osf.io/q56ns. ERPs are typically generated by the repeated presentation of transient stimuli while the EEG is recorded, often at temporal intervals of several seconds. Stimulus‐locked EEG segments obtained for each presentation are then averaged, resulting in a prototypical voltage time course (David, Kilner, and Friston 2006; Di Russo et al. 2002). By contrast, ssVEPs result from rhythmic presentation of visual stimuli (e.g., regularly flickering light), driving an oscillatory neural response at the same temporal frequency as the driving stimulus (Norcia et al. 2015; Wieser, Miskovic, and Keil 2016).

Data set 1 comes from a picture viewing task in which ERPs were measured from 39 participants (27 women; average age 19.6 years) while passively watching pleasant, neutral, and unpleasant affective pictures from the international affective picture system (IAPS) (Lang and Cuthbert 2008). The present demonstration focuses on two conditions, pleasant and unpleasant picture viewing. These conditions were selected because they each prompt robust ERP signals but tend to show only small condition differences when compared using univariate methods (Gibney et al. 2020). A total 20 pleasant and 20 unpleasant pictures were shown twice each, for a duration of 1 s at the central portion of the screen, resulting in a total of 80 trials per participant, with 40 trials in the pleasant and 40 in the unpleasant condition.

Data set 2 comes from a fear conditioning paradigm in which neutral cues were flickered at 15 Hz, prompting an oscillatory SSVEP response, recorded in 31 participants (22 women; average age 19.23 years). Visual cues consisted of 4 high‐contrast circular sinusoidal gratings (orientations: 15°, 35°, 55°, and 75°) filtered with a Gaussian envelope (Gabor patches). Each stimulus was presented against a dark gray background in a flickering (15 Hz) mode for 3 s. The fear generalization protocol (Lonsdorf et al. 2017) involved three phases: First, in the habituation phase, each of the 4 visual cues was presented 30 times in random order. Later, during the fear acquisition phase, the 4 visual cues were again shown, but one of them (CS+, 15° or 75° randomly assigned per subject) was followed by an aversive loud (88 or 91 SPL dB) white noise (the unconditioned stimulus, US) that started 2 s after the onset of the CS for a duration of 1 s, thus co‐terminating with the CS. Finally, in the extinction phase, visual cues were presented to the participant again, but no US was presented any time. After acquisition, the CS+ orientation typically acquires aversive properties. Here, we compare the neural activity response to the CS+ visual cue in the habituation and the extinction phases.

Procedures of both experiments were approved by the University of Florida institutional review board and informed consent was signed by all participants in accordance with the Declaration of Helsinki.

#### 2.1.1 EEG Data Acquisition and Preprocessing

EEG signals from both datasets were collected using an Electrical Geodesics (EGI, Eugene, OR) system with a 128‐channel HydroCel electrode net, and continuously digitized at 500 Hz, referenced to the vertex sensor (Cz). Electrode impedances were kept below 50 kΩ. Online (low‐pass filter, 3 dB point at 170 Hz; high‐pass filter, 3 dB point at 0.05 Hz) and offline (low‐pass filter, 40 Hz, 18th order, 3 dB point at 0.05 Hz) Butterworth filters were applied to the data. Then, the signal was segmented in epochs including 0.2 s pre‐stimulus and 1 s post‐stimulus for the ERP and 1 s pre‐stimulus, and 2 s post‐stimulus for the ssVEP datasets. Statistical Correction of Artifacts in Dense Array Studies (SCADS) procedure (Junghofer et al. 2000) was employed for artifact rejection of bad channels and epochs. Finally, all trials were individually baseline corrected by subtracting the voltage activity average in the pre‐stimulus segment of the same trial and ordered in categories pleasant (1037 trials across all participants) and unpleasant (1133 trials) for the ERP dataset and CS+ (548 trials), GS1 (570 trials), GS2 (571 trials), GS3 (578 trials) for habituation, and acquisition phases of the ssVEP dataset.

#### 2.1.2 Current Source Density (CSD)

EEG recordings are characterized by pronounced spatial smoothing and distortion as signals travel from the brain to the extracranial sensor locations, caused by a number of biophysical processes including volume conduction and spatial lowpass filtering. The reduction of shared information is crucial in terms of assessing the spatial generalization of time‐based decoders (Keil et al. 2022). Here we applied the CSD transformation, an established algorithm for diminishing spatial smearing in the topographical distribution of EEG signals (Nunez et al. 1997). Specifically, we used the implementation by (Junghofer et al. 1997) (Gibney et al. 2020) setting the smoothing (regularization) parameter *λ* to a value of 2.

### 2.2 Decoding Analysis for Brain Response Generalization Across Spatial and Time Dimensions

#### 2.2.1 Electrophysiological Waveform‐Based Classification

Time‐GAL first applies (trains) a linear discriminant decoder to discriminate two conditions based on temporal features of the electrophysiological signal. Then, in a second step, it quantifies the generalization of the temporal decoders across channels, a process known as cross‐decoding. This approach is similar to the GAT method (King and Dehaene 2014), where a spatial classifier is applied across different time points. Time‐GAL implements the GAL approach where each channel's temporal decoder is tested at all other channel locations, a process known as cross‐decoding. Because this decoding approach aims to infer neural processes from data, it can be considered a backward model (Haufe et al. 2014). In contrast, a forward model is a model that describes how brain activity results in neural data as collected for example by EEG. Because there is a corresponding forward model to each backward model (Haufe et al. 2014; David, Kilner, and Friston 2006), the time‐GAL approach can employ a forward modeling approach to obtain the information regarding when each topographic channel reflects condition differences.

**FIGURE 3:**
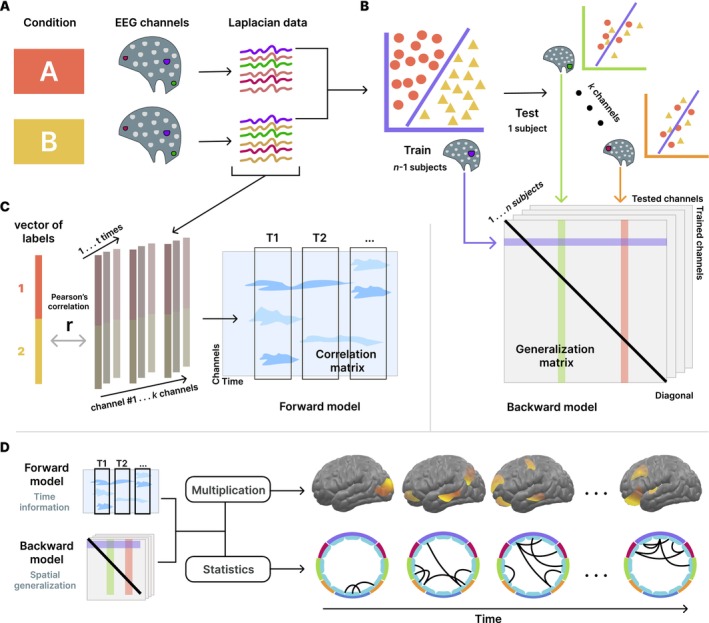
Flowchart describing the decoding methodology based on the temporal and spatial information. (A) Laplacian EEG data from conditions A and B are used to train one classifier per channel using time points as features. (B) Next, the generalization matrix (backward model) is calculated by testing every classifier model with data from each of the remaining channels (cross‐decoding). (C) Then, the correlation matrix (forward model) is calculated using Pearson's correlations between the A/B labels and EEG data for each channel and time. (D) Finally, the combination of both backward and forward models containing the spatial and temporal information, respectively, allows us to visualize the GAL cross‐decoding patterns in the brain. We interpret these as similarity or connectivity matrices, akin to correlation‐based connectivity matrices used in EEG and fMRI research.

#### 2.2.2 Backward Model of the Generalization Across Location

For the GAL analysis, we use data from two conditions, referred to as A and B. In our implementation, each data set consisted of three‐dimensional arrays (channels by time points by trials) for each condition and each subject (see Figure 3 top left). For one of *k* channels, we fed the classifier model all time points as features and all single trials from all *n* participants except from one participant (leave‐one‐out training/testing), as observations. Because the classifier operates at the single trial level, many observations were considered for the training phase of the classifier model, creating pressure on computing resources. Therefore, we compared the performance and efficiency of a linear discriminant analysis (LDA) with a support vector machine (SVM). LDA is proven to be suitable for neural decoding (Mandelkow, De Zwart, and Duyn 2016), although SVM has been more widely used in EEG data (Bae and Luck 2019, 2018) for averaged ERPs. Similar to Grootswagers, Wardle, and Carlson (2017), we found comparable decoding rates from both types of models but a significant reduction of time consumption from the LDA algorithm. Because we perform a substantial amount of classifier training and we seek a computationally efficient methodology, we selected LDA as our classifier model to discriminate training trials as coming from conditions A or B. A performance comparison among LDA, SVM, and multi‐layer perceptron (MLP) classifier models is provided in Appendix S2. Models were trained using the LDA function built in (The MathWorks Inc. 2023). Even when the number of trials were unbalanced, all single trials from conditions A and B were employed to avoid randomness in any aleatory selection of trials. Priors were set accordingly. Information contained at single‐trials is noisier than evoked information from trial‐averages (e.g., ERPs), but single‐trial time courses include the non‐phase‐locked temporal features of the signal, often referred to as “induced”, instead of “evoked” responses (David, Kilner, and Friston 2006). More information about the difference between applying the GAL methodology to single‐trial or averaged is given in Appendix S1.

In the second step of the time‐GAL method, we applied the classifier model weights trained at a given channel to quantify its decoding performance with data from all *k* channels using the single trials of the remaining subjects as the test data set. Thus, we required the decoder to use feature weights that were applicable across participants. The GAL test phase provides a classification of trials into A and B that quantifies the accuracy of the model using the decoding accuracy (correct A and B classification) as an index. This procedure was repeated *k* times, training the classifier model with all the remaining channels and testing their weights with all the *k* channels to fill out the decoding accuracy in a generalization matrix of training by testing axes with dimensions *k* by *k* channels (see Figure 3 middle right). Finally, this analysis was computed again for each subject, using the rest of the *n* − 1 participants as training dataset in a leave‐one‐out approach. Thus, this approach to cross‐validation avoids arbitrary decisions (e.g., regarding the number of k‐folds) in the training/test data set selection. Finally, this methodology provides us with *n* generalization matrices of channel‐by‐channel dimension, where *n* corresponds to the number of subjects. This distribution is employed later in the following statistical analyses. Conceptually, if a time‐based decoder trained at location *k*_i_ also classifies the data at location *k*_j_, then we interpret this as evidence that the temporal dynamics at *k*_i_ and *k*_j_ contain share decodable information about the conditions—a non‐parametric way of describing spatial dependency or connectivity.

#### 2.2.3 Forward Model of the Temporal Contribution

The GAL method allows us to inspect the spatial distribution of the decoding performance between A and B conditions, based on the electrophysiological waveform, or time information. However, this approach offers only a static picture of the connectivity matrix. In the GAL framework, a “connection” indicates that two channels share decodable information. Therefore, it is helpful to visualize what time points in the waveform are most strongly related to the discrimination of conditions. As stated above, it is possible to estimate a forward model to obtain the weights of the LDA features (Haufe et al. 2014). We can address this estimation by measuring the relations between the condition labels and the Laplacian EEG data. To this end, the Pearson's correlation coefficient between the true vector of A and B labels and all subjects' trials of electrophysiological activity was calculated for each time point and channel resulting in a channel by time matrix of correlations (see Figure 3 middle left).

#### 2.2.4 Combining Decoding Topography With the Time Domain

While the backward model provides spatial information, we use the forward model to visualize the temporal progression of condition differences at the single trial level. Specifically, we combine the information from both models to obtain a temporally weighted representation of the decoding patterns. First, we were interested in a brain topographic visualization of the main contributor areas to the decoding analysis. To obtain this visualization, we used the diagonal vector from the average of the *n* generalization matrices, that is, the decoding accuracy of each channel's LDA, across participants. This information served to project the decoding rates of the *k* channels into the source space. Then, the decoding‐weighted forward model is obtained by timepoint‐wise multiplication of the overall decoding vector of *k* spatial values with the absolute values of the *k* by *time* correlation matrix. The absolute value of the correlation matrix is used because it indicates the contribution of a given channel and time point to the discrimination between conditions A and B, regardless of the sign. This combination of the decoding vector (spatial information) and correlation matrix (temporal information) results in another *k* by *time* matrix, which reflects the time‐weighted spatial contribution to the decoding. Thus, we obtain one topography that represents the spread of decodable information across the brain surface for every *time* step. EMEGS 2.8 (Peyk, De Cesarei, and Junghöfer 2011) was used to project this information onto a standard cortical surface, for visualization.

#### 2.2.5 Statistical Extraction of GAL Connectivity Patterns Over Time

For the statistical analysis of GAL data, a mass univariate approach was chosen. Once the *n* generalization matrices were computed, Student's *t*‐test against the decoding chance rate were computed for each element in both directions, that is, above and under the chance level. As we only have conditions A and B, the decoding chance rate of the classifier is 0.5. The diagonal of the GAL matrix shows the decoding accuracy for the test dataset of the same channel used for classifier training, and thus its values are not expected to be smaller than chance, that is, 0.5. However, the values for the rest of the generalization matrix, in which test and training datasets came from different channels may well show values below chance level. This would indicate that the data from one location are systematically located on the other side of the hyperplane spanned by the LDA trained on another location. This may be interpreted as those locations showing orthogonal neural dynamics, more like the other label, resulting in significantly below‐chance decoding. Therefore, we used the contrast *t*‐test in both directions showing not only those positive connections between regions, but also negative connections (below chance cross‐decoding). Each of these analyses resulted in one matrix of channel‐by‐channel dimensions showing a large number of comparisons. As all the connectivity values of the matrix derives from *k* decoders (i.e., the same number of channels) and is using *k* different timeseries, only dependency across rows and columns can be assumed. Therefore, Bonferroni was applied to address the multiple‐comparison problem assuming *k* independent sources. Also, to obtain only the most meaningful generalization connections, a corrected alpha (α) was utilized. By default, the time‐GAL toolbox uses a α value of 0.05. Therefore, only connections with a p‐value less than $\frac{\alpha }{k}$ where α represents the desired alpha level, were considered statistically significant and used for the description of the connectivity (GAL) pattern.

For visualization, in a final step, we combined the statistically significant GAL results with the statistically significant temporal correlations to illustrate the time‐GAL dynamics. Specifically, we only considered the Pearson's correlation significance of those channels that showed statistically significant generalization connections (see above). We used a threshold of *α* = 0.01 (set as default in the toolbox) to identify temporal correlations (contributions) of each location that were relevant for visualization. This alpha value was not Bonferroni corrected because it was only used as a threshold for visualization. This step allowed us to visualize the relationship between areas throughout the temporal progression of the neural information processing. Thus, we can inspect how different networks or areas are implicated in different time steps, providing a descriptive video of the cognitive processing instead of a static picture of all the GAL pattern.

Finally, visualization of decoding results at the cerebral space was performed using a source‐reconstruction model. To this end, the Brainstorm suite of applications (Tadel et al. 2011) was used to create a brain mesh model and to compute the minimum‐norm source reconstruction of the neural activity (Gramfort et al. 2010). Note that this topographical visualization was implemented in the toolbox to present the results of the example datasets, and is only valid for data collected in the 128‐channel HydroCel electrode net (Electrical Geodesics, EGI, Eugene, OR).

## 3 Results

The time‐GAL toolbox uses a classification approach to quantify decodable temporal information in neural dynamics, including how this information generalizes across locations. In the following, we first illustrate how the time‐GAL methodology captures information contained in the ERP response to transient visual stimuli. Later, we examine the application of time‐GAL to ssVEP responses.

### 3.1 Time‐GAL Patterns in ERP Waveforms: Affective Picture Presentation

The first dataset consists of CSD‐transformed EEG recordings obtained during the presentation of pleasant and unpleasant pictures from a standardized picture database, in a within‐participants design. The pipeline of this example is detailed in a MATLAB live script called “Analysis_Dataset1_ERP.mlx” accessible from the GitHub repository (https://github.com/csea‐lab/time‐GAL) and OSF repository (https://osf.io/q56ns). However, the example dataset itself is only accessible from the OSF repository (folder “Dataset1_IAPS_ERP”). This folder contains 78 files belonging to each subject (*n* = 39) and condition (“Data_Pleasant”, “Data_Unpleasant”). The time‐GAL toolbox requires data to be formatted in one array for each condition, with dimensions channel, time, and trials. Therefore, the toolbox function “groupCondition()” collects data from each participant's data file for a given condition in the dataset folder and groups the information in a single three‐dimensional array. Additionally, the function generates a vector that specifies the subject associated with each trial (see Figure 2). This function is independently run for pleasant and unpleasant conditions, thus generating two data arrays and two vectors of subject identifiers that are input into the time‐GAL function.

In the next step, we used the time‐GAL toolbox to perform pairwise decoding of the two conditions (pleasant vs. unpleasant). The GAL approach was computed (function “timeGAL()”) using only the channels of interest (see Section 2) and using the MATLAB built‐in Parallel Computing Toolbox with 4 cores to increase processing speed. A Bonferroni‐corrected *α* of 0.05 (set by default in the toolbox) was used as the statistical threshold for the generalization connectivity patterns.

**FIGURE 4:**
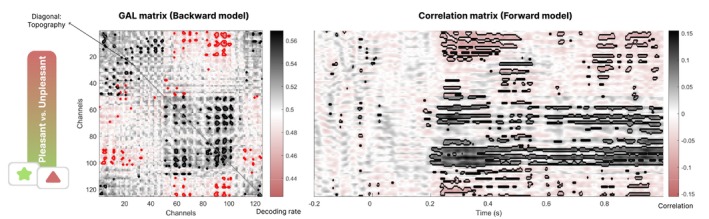
Generalization connectivity and temporal distribution matrices for the affective picture viewing experiment. Left: Generalization connectivity matrix showing decoding results (accuracy). The y‐axis corresponds to the channel data employed to train the model, and the x‐axis shows the channel data used for the generalization test. Black and red contours indicate the positive and negative generalization connections, respectively, after a Bonferroni‐corrected statistical (*α* = 0.05) comparison against chance (50%). Right: Temporal correlation between labels of condition and data showing the positive or negative weights of the decoding model. Black contours indicate the time points whose *p*‐value is below 0.01 for each channel.

**FIGURE 5:**
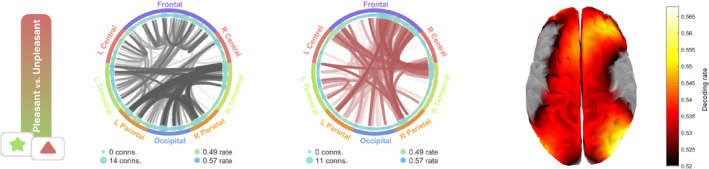
Generalization connectivity patterns and topography for the affective picture viewing experiment. Left and middle: Connectivity graph showing the significant generalization connections between the CSD channels. Black lines refer to connections in which both channels share decodable temporal patterns, while red lines (Middle) show the opposite pattern (significant below chance decoding). Right: Topographical representation of the decoding rate of the diagonal of the GAL matrix, that is, the discriminative capability of each sensor to decode between pleasant and unpleasant conditions. A source reconstruction method was used for brain space representation of the decoding rate.

**FIGURE 6:**
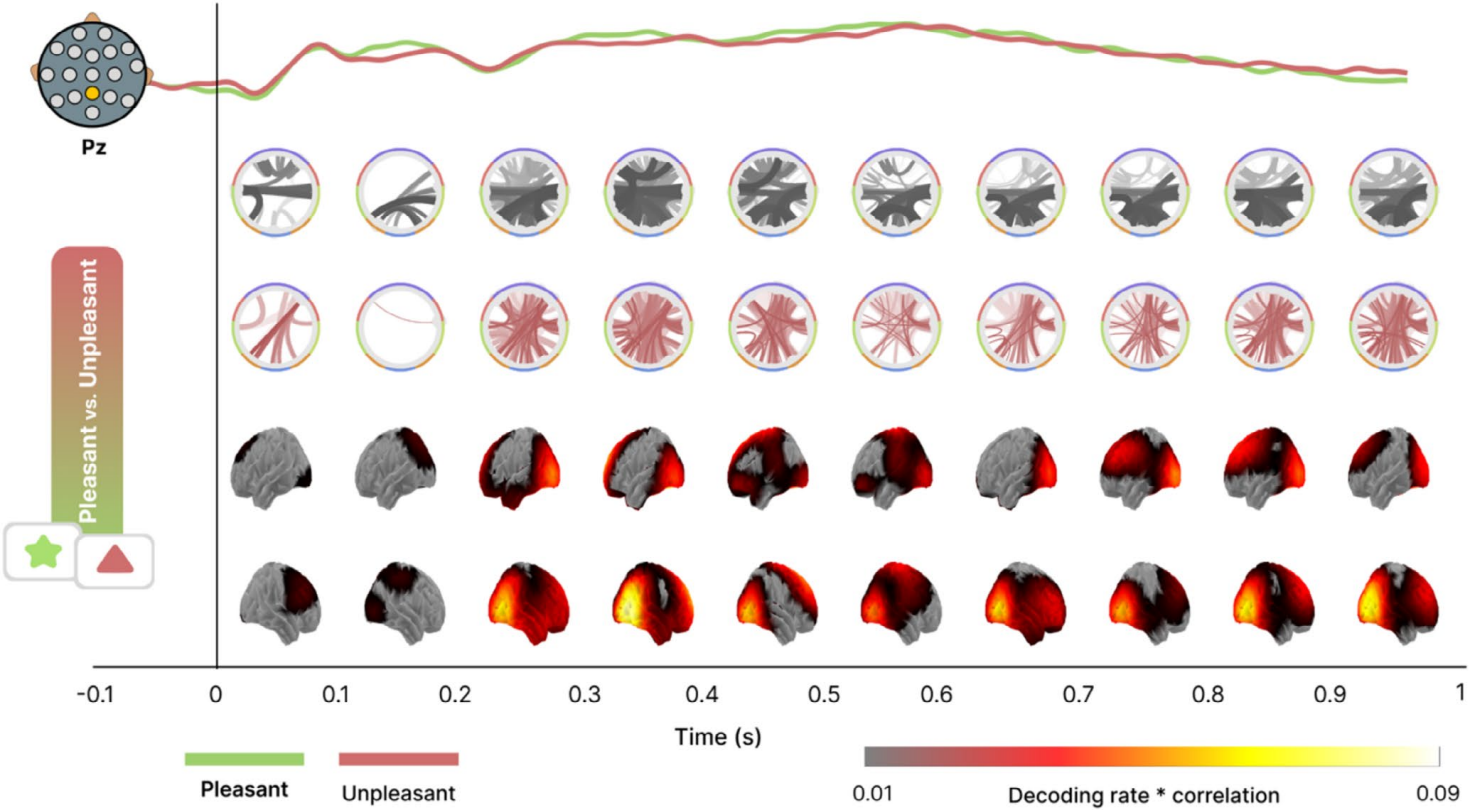
Time course of emotional content decoding and GAL connectivity patterns during affective picture viewing. Top: Electrophysiological ERP response to the presentation of different emotional content obtained at the parietal central (Pz) electrode for the two conditions (pleasant and unpleasant). Middle: Circular graphs show the GAL connectivity pattern at each time step for each pairwise condition. The topographies (Bottom panel) indicate the multiplication of the decoding rate with the time‐varying correlation, showing changes in cross‐decoding across the surface of the brain as a function of time.

Once the decoding analysis is completed, all results can be displayed by feeding the time‐GAL output into the toolbox function called “visualizationTimeGAL()”. This function plots three views of the decoding results: backward and forward model matrices (Figure 4), connectivity and topography (Figure 5), and time course of connectivity and topography (Figure 6). The first plot, the GAL (backward model) and correlation (forward model) matrices, represent the main output of the toolbox and are shown in Figure 4.

The GAL matrix represents the pattern of statistically significant connectivity links between channel locations. The resulting configuration relies on the temporal patterns, that is, features of the model, found in the data. The correlation matrix represents a forward model and provides information about the temporal patterns discriminating the experimental conditions. In the example data, correlations between condition labels and channel and time points indicate the emergence of decodable information around 250 ms after trial onset.

Then, from the GAL matrix (located in the variable “timeGALoutput.GeneralizationMatrix.GAL”), we can visualize the connectivity pattern using the function “circularConnectivity125static()”. Its diagonal provides the *k* values to plot the temporal decoding topography (variable “timeGALoutput.GeneralizationMatrix.Topography”). The resulting connectivity patterns and topography are shown in Figure 5.

Several posterior channels showed significant decoding based on their time dynamics. Similarly, significant generalization connections were more concentrated among posterior areas. It is worth noting that inverse connections (below chance decoding, in red) were observed between anterior and posterior regions, likely representing opposite voltage of dipolar fields when measured at opposite sides of the head.

Next, we combined the spatial information of the generalization response across the brain with the temporal weights of the model for each channel. Specifically, we multiplied the diagonal of the generalization connectivity matrix with the Pearson's correlation matrix of time weights. Additionally, we determined which generalization connections and temporal weights were statistically significant. This topography and connectivity across time can be found in the time‐GAL toolbox output in variables “timeGALoutput.TimeGAL.TimeTopography” and “timeGALoutput.TimeGAL.TimeGAL”, respectively. As shown in Figure 6, both sources of information shed light on cortical surface regions that contain decodable information (brain surface plots, bottom panels) and how they share decodable information (circular graphs).

The time course analysis showed strong inverse (below‐chance) decoding between posterior and anterior regions and several direct (positive) generalization connections between right occipital‐temporal and parietal zones. The fronto‐occipital connectivity pattern was present from 0.2 to 0.5 s and at the end of the trial from 0.7 to 1 s, while right occipito‐temporal generalization persisted from 0.2 s to the end of the trial. As discussed above, the inverse direction of the GAL results in the antero‐posterior connections likely indicates that the voltage patterns found in the occipital and parietal areas are inverted at frontal sensors prompting below chance decoding accuracy. By contrast, the right parietal time‐GAL patterns are consistent with studies of the late positive potential as mentioned below in the discussion section.

### 3.2 Time‐GAL Patterns in ssVEP Signals: Aversive Conditioning of Oriented Gabor Patches

**FIGURE 7:**
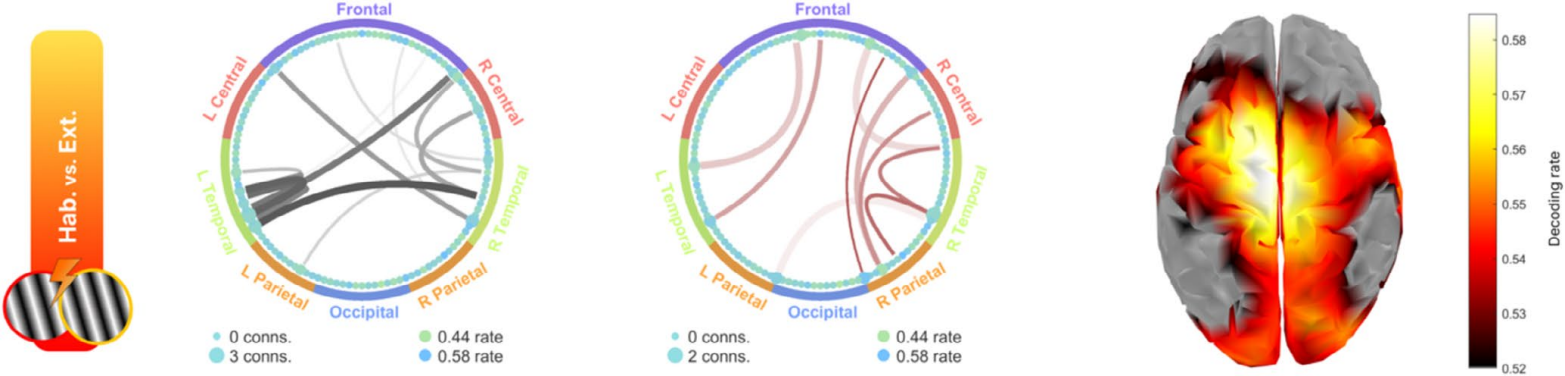
Generalization connectivity patterns describing changing temporal dynamics during the course of fear conditioning and extinction. Left and middle: Circular graphs showing the statistically significant generalization connections between 125 CSD channels. Black lines refer to connections where both channels show similar decoding temporal patterns, while the graph with red lines (middle) indicates channels showing inverse decoding patterns. Right: Topographical representation of the decoding accuracy for the CS+ stimulus when viewed during the habituation versus the extinction phase.

Next, we applied the timed‐GAL approach to ssVEP data from an experiment using flickering visual stimuli to compare the neural response to the CS+ (threat cue) stimulus between the habituation (pre‐acquisition) and extinction (post‐acqusition) phases of the experiment. Here, we followed the same procedure as used in the previous example. The corresponding time‐GAL analysis pipeline can be found in the MATLAB live script named “Analysis_Dataset2_ssVEP.mlx”, and the data may be retrieved from the folder “Dataset2_FearConditioning_ssVEP” on the OSF repository. Results presented below in Figure 7 use data from the habituation and extinction phase of the fear conditioning task, before (habituation) and after (extinction) the CS+ stimulus (a grayscale grating defined by its orientation) became a threat cue due to consistent pairing with an aversive white noise (US) during the intermediate acquisition phase.

In terms of direct generalization connections (black lines in Figure 7), the habituation versus extinction comparison of the CS+ response shows shared decodable information across both temporal and central areas. Regarding inverse generalization (red lines in Figure 7), generalization was observed ranging from parietal to temporal, central and frontal areas, especially in the right hemisphere. The topography shows a concentration of the decoding rate along the visual cortices and the superior central area. Such areas could be related with the visual processing of the CS+ and motor preparation response associated during the acquisition phase.

**FIGURE 8:**
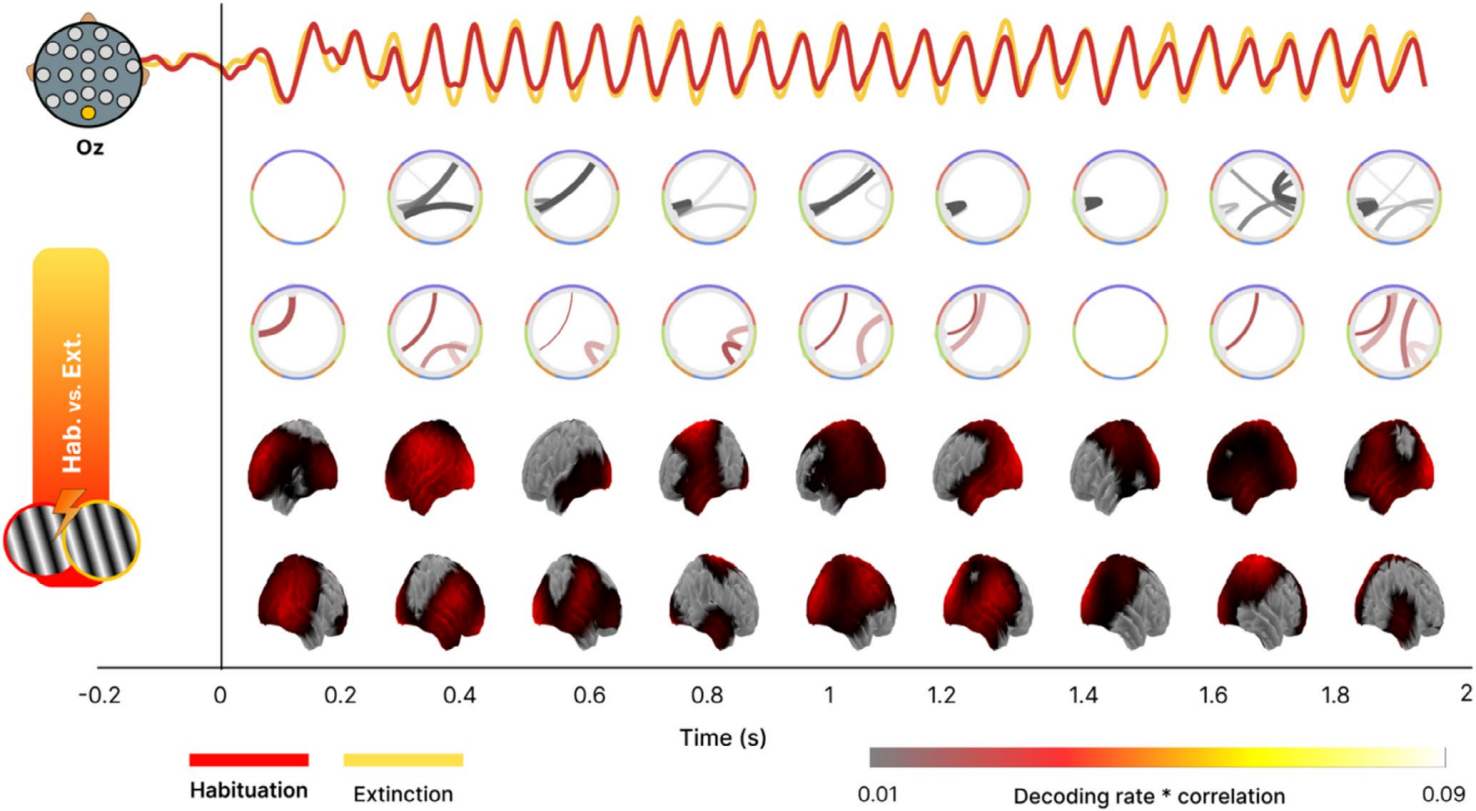
Time course decoding and GAL connectivity patterns applied to a fear conditioning experiment. Top: Electrophysiological ssVEP response to the presentation of CS+ cues obtained at the Occipital central (Oz) electrode for the habituation and extinction phases. Middle: Circular graphs show the direct (black lines) and inverse (red lines) GAL connectivity pattern at each time step. Topographies (bottom) depict the time dynamics of decoding strength across the cortical surface.

Next, we explore the time‐GAL and associated topographies to visualize how the ssVEP evoked by the CS+ visual cue is modulated as result of fear conditioning. Figure 8 shows these time dynamics.

Here, we found that the connectivity and topographical modulations largely take place immediately after stimulus presentation, and toward the end of the trial, just before onset of the aversive noise (US). Specifically, direct connectivity between temporal and frontal areas appeared during the first part of the trial (0.2–0.4 s), and then a more widespread connectivity pattern appeared toward the end of the trial (1.6–2 s). Regarding inverse connectivity, temporo‐frontal links were seen throughout the trial.

Taken together, both the GAL connectivity and the decoding topography show two temporal windows of interest. A more posterior, initial, effect between 0.2 and 0.4 s, and another between 1.8 and 2 s–the time at which the US was presented during the acquisition phase. In this second time range, the time‐GAL analysis suggested involvement of visual, motor and frontal cortices, consistent with expectancy and defensive mobilization.

## 4 Discussion

The emergence of multivariate decoding techniques has made it possible to quantify and discriminate dynamic changes in neural patterns. Here, we present a decoding toolbox that first quantifies the temporal patterns in neural population signals that are unique to a given condition. The time‐based decoder is then used to quantify the similarity of time courses across different locations. Furthermore, the combination of this decoding approach with a forward model visualizes the changing cross‐decoding over time and thus illustrates the temporal sequence of spatial activation patterns.

The present report demonstrates the usage of the Time‐GAL toolbox in a step‐by‐step fashion, employing two EEG datasets recorded during two tasks, both involving vision and emotion. In the first study, consistent with a large body of evidence (Liu et al. 2012), we found strong decoding during the period of the late positive potential (LPP) ranging from 0.5 to 1 s following the onset of the picture stimuli. However, previous univariate studies rarely observed differences between pleasant and unpleasant content, supporting the notion that the multivariate time‐GAL approach is more sensitive than univariate approaches. In addition, while univariate models (Friston, Jezzard, and Turner 1994) have occasionally identified condition differences between pleasant and unpleasant content during the LPP time window, the MVPA‐based approach extracted the spatial pattern differences and highlighted brain areas with shared decodable information. Specifically, dense connectivity within posterior areas and anterior areas were found in different time windows, suggesting that diverse perceptual and emotional processes take place during the trial. Therefore, the time‐GAL method has substantial potential for refining spatio‐temporal analyses of emotional scene perception.

The application of time‐GAL to the aversive conditioning dataset (data set 2) illustrated how the method discriminated between neural responses to the threat‐associated visual cue before and after conditioning. We found differences in the neural response to the same cue (CS+) between both phases, highlighting the sensitivity of the method to acquired stimulus properties such as conditioned threat (Lonsdorf et al. 2017). In our example, the generalization connectivity was most pronounced during early perceptual analysis and then again toward the end of the trial, where a US may be expected. The decoding topography dovetailed with the existing literature (Moratti and Keil 2005) highlighting the involvement of visual and motor areas in visual discriminant fear learning.

Instead of using spatial information as traditionally carried out in fMRI (Haxby 2012; Peelen and Downing 2023) and M/EEG (Ashton et al. 2022; Bae and Luck 2019, 2018), here we utilize the temporal dimension as features to feed into the decoding model. Previous work (Bode et al. 2014, 2012, 2019) has already used temporal information in MVPA models, for example by splitting the signal in small windows. In contrast, our approach does not only use the entirety of the time information for decoding, but also employs it in a forward model to describe the dynamics of decodable information. Therefore, the present toolbox may represent a much needed step toward spatio‐temporal characterization of neural activity patterns while taking advantage of their high temporal resolution.

Decoding approaches have been applied to neuroimaging data in different ways. Bae and Luck 2018 have used time‐resolved MVPA on averages of trials to achieve better signal‐to‐noise ratios and to reduce computational resources. This approach amplifies the time‐locked information contained in the trials, and attenuates the non‐time‐locked (induced) information of each individual trial (David, Kilner, and Friston 2006). Following their approach, we computed a version of the GAL procedure making use of averaged groups of trials (see Appendix S1). Overall similar information was found in the generalization connectivity matrices using averaged data compared with the single‐trial based method proposed in this paper. Interestingly, no statistically significant inverse generalization connections were found when using trial averages, indicating that the non‐phase‐locked activity may contribute to single‐trial based decoding.

In past decades, various MVPA toolboxes have emerged in the field of electrophysiology, offering new and powerful decoding‐based methodologies for neuroimaging analysis (Fahrenfort et al. 2018; López‐García et al. 2022; Treder 2020; Lu and Ku 2020). However, most of them offer a standard decoding approach, using spatial information as features for the classifier model. This approach enables the computation of the GAT (King and Dehaene 2014), but often does not include quantifying the spatial features underlying the decoding. To our knowledge, only the Decision Decoding ToolBOX (DDTBOX) (Bode et al. 2019) offers the possibility to specifically study time series as features in MVPA analyses. Other approaches use spatio‐temporal data and feature similar capabilities, for example through weight mapping or permutation‐weight methods (Chard et al. 2024; Csaky et al. 2023). These latter algorithms may also be used on connectivity matrices (Giovannetti et al. 2021). Compared with these approaches, the time‐GAL toolbox first isolates decodable information that is purely temporal in nature. It then advances existing approaches by implementing cross‐decoding and by allowing the combination of both forward and backward models. This integration allows researchers to characterize the spatio‐temporal particularities of the input data.

The proposed approach can be easily applied using the Time‐GAL toolbox available in https://github.com/csea‐lab/time‐GAL. No statistical assumptions are required to be met and both stationary and non‐stationary signals can be used for decoding. Therefore, this procedure can be utilized to analyze a wide range of cognitive and affective neurophysiological studies. In the present study, a current source density transformation was applied to minimize effects of volume conduction in EEG data, but this step is not required. Also, the use of a LDA classifier model instead of more computationally heavy algorithms such as SVM or neural networks (e.g., MNEFlow toolbox (Zubarev et al. 2019)) offers to the researcher a relatively fast approach to compute the training/test calculations for the decoding analysis (see Appendix S2). This toolbox was created in the MATLAB environment, a widely used environment for neuroscience researchers and it does not require the use of graphical card for accelerated computation. More details about the correct use and properties of the toolbox can be found in its documentation: https://github.com/csea‐lab/time‐GAL/wiki or https://osf.io/q56ns/wiki/home/.

The second step of the time GAL procedure offers information about how decodable information is generalized across the brain. The generalization connectivity maps obtained through the algorithm may be suitable to examine the configuration of dynamic cortical networks (Pessoa 2014). Traditionally, these networks have been studied conducting functional connectivity analysis using measures that reflect the statistical dependencies between two time series, such as the Pearson correlation coefficient or other metrics derived from general linear models (Friston, Jezzard, and Turner 1994). This methodology is often applied to resting‐state recordings (López‐Sanz et al. 2017) and tends to involve filtering of the data to highlight a given aspect of the neural time series. Here we extract decodable information from the waveform itself at the single trial level, using all available data. Hence, the Time‐GAL toolbox provides a decoding‐based approach for examining the configuration of the cerebral connectivity during a cognitive process, representing a novel feature with respect with the rest of decoding toolboxes.

In conclusion, time‐GAL is a novel algorithm for examining neural dynamics based on decoding. At its core, it is non‐parametric in nature because it is based on decoding accuracy measured by leave‐one‐out cross‐validation. Thus, the toolbox has substantial potential for application to wide range of spatio‐temporal biological signals, especially signals characterized by rich temporal information.

## Supporting information


**Appendix S1.** Supporting Information.

**Appendix S2.** Supporting Information.

## Data Availability

The data that support the findings of this study are openly available in Time‐GAL toolbox at https://osf.io/q56ns/, reference number 10.17605/OSF.IO/Q56NS.
