# Supplementary material for: Decoding in the Fourth Dimension: Classification of Temporal Patterns and Their Generalization Across Locations
Source: Hum Brain Mapp. 2025 Jan 30;46(2):e70152. doi: 10.1002/hbm.70152 (PMC11780319; doi:10.1002/hbm.70152)
Supplement: Supplementary file 1 — Appendix S1. Supporting Information. [file HBM-46-e70152-s001.pdf]

Supporting Information 1 Appendix

Traditionally, the MVPA-based decoding approach has been conducted using spatial information to classify between conditions [1]. Usually, information from the experimental trials is condensed in averaged groups of trials to reduce the computational resources needed to train the model [2,3]. Therefore, classifier models requiring heavier computational resources, such as supported vector machine (SVM), can be used. However, in our work we employed linear discriminant analysis (LDA), a classifier method requiring less resources, thus allowing us to compute the generalization across location (GAL) methodology at the single-trial level.

Analysis of brain electrophysiological data following an evoked (averaging data) or induced (using single-trials) approach can derive different results [4,5]. For this reason, here we aimed to investigate whether the results of the GAL methodology using an evoked one similar as performed by Bae and Luck (2018) could differ from the induced approach developed in the main text. Particularly, in this complementary analysis we randomly divided the trials for each condition in three averaged groups using two for training the model and one for the test phase. Again, a leave one subject out cross validation was carried out training the model using *n*-1 subject and testing in the remaining subject. For simplicity and better comparison, ERP signal from the affective EEG task was employed since it benefits better from average than SSVEP signal.

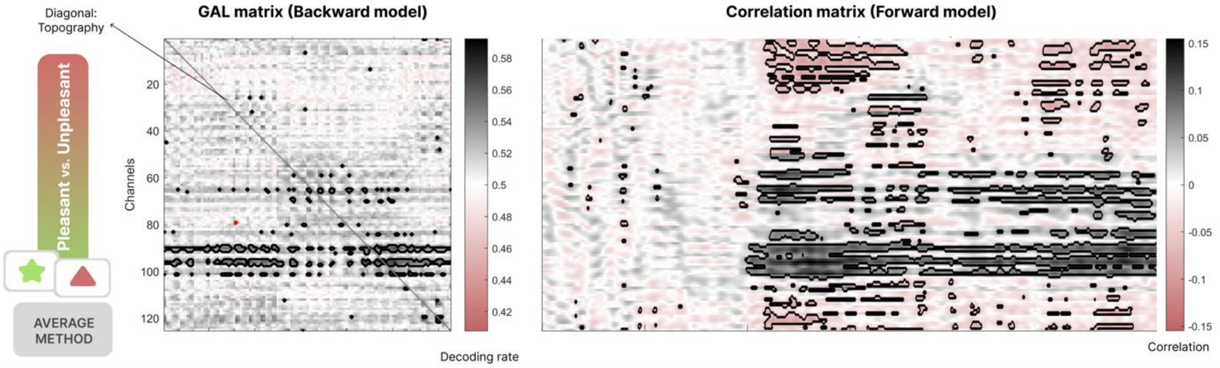

Figure S1.1. Generalization connectivity and temporal distribution matrices for the affective picture using an average-based evoked approach. **Left**: Generalization connectivity matrix showing decoding results (accuracy). The Y axis corresponds to the channel data employed to train the model, and the X axis shows the channel data used for the generalization test. Black and red contours indicate the positive and negative generalization connections, respectively, after a Bonferroni-corrected statistical (α = 0.05) comparison against chance (50%). **Right**: Temporal correlation between labels of condition and data showing the positive or negative weights of the decoding model. Black contours indicate the time points whose p-value is below 0.01 for each channel.

Critically, we can observe a almost a lack of inverse generalization connectivity (in red), indicating that the evoked method does not find statistically significant neural patterns of opposite response to the stimuli. This difference with respect to the evoked methodology may highlight the capability of the MVPA tools to extract the information from single-trials contained at the induced level and lost when information is averaged.

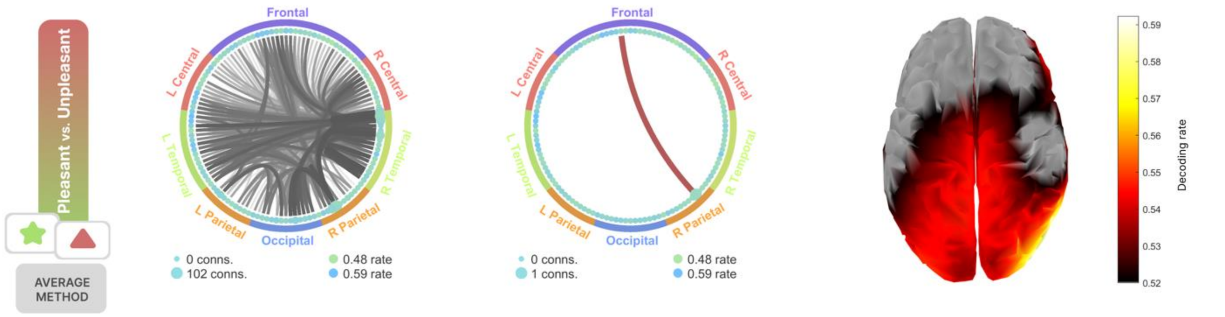

Figure S1.2. Generalization connectivity patterns and topography for the affective picture viewing experiment using an average-based evoked approach. **Left and middle**: Connectivity graph showing the significant generalization connections between the CSD channels. Black lines refer to connections in which both channels share decodable temporal patterns, while red lines (Middle) show the opposite pattern (significant below chance decoding). **Right**: Topographical representation of the decoding rate of the diagonal of the GAL matrix, i.e. the discriminative capability of each sensor to decode between pleasant and unpleasant conditions. A source reconstruction method was used for brain space representation of the decoding rate

Regarding the generalization connectivity patterns (Figure 5 left and Figure S1.2 left), both analysis shows slight differences but preserving the same anterior-posterior generalization connectivity patterns. Inspecting the decoding topographies (Figure S1.2 right), we can observe similar decoding patterns than those obtained using an induced methodology (Figure 5 right). However, involvement of medial frontal areas and the frontal pole shown in the induced method does not appear in the evoked one. These results suggest the involvement of oscillatory-dependent activity in higher order processing areas such as frontal cortices.

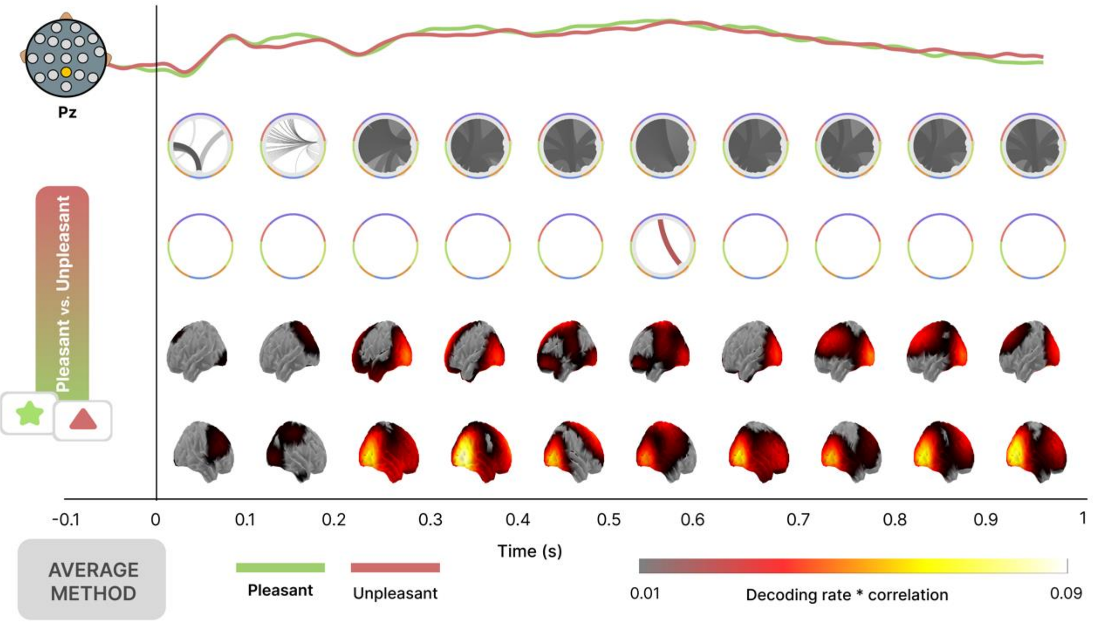

Figure S1.3. Time course of emotional content decoding and GAL connectivity patterns during affective picture viewing using an average-based evoked approach. **Top**: Electrophysiological ERP response to the presentation of different emotional content obtained at the parietal central (Pz) electrode for the two conditions (pleasant and unpleasant). **Middle**: Circular graphs show the GAL connectivity pattern at each time step for each pairwise condition. The topographies (**Bottom** panel) indicate the multiplication of the decoding rate with the time-varying correlation, showing changes in cross-decoding across the surface of the brain as a function of time.

Regarding the time-GAL results (Figure S1.3), similar anterior and posterior distribution of the neural patterns are found during the main part of the trial (from 200 ms to 1 s). Nevertheless, more occipito-frontal connectivity was shown in the last part of the trial (800 ms to 1 s). Accordingly, topographies during the last part of the trial corresponds with this occipito-frontal interaction. Compared with the evoked results (Figure 6), both latencies and topographies show similar. Main differences between both procedures can be found in the cerebral connectivity patterns, overall due to the above-mentioned lack of inverse generalization connectivity.

In general, results from the evoked time-GAL procedure highlights the role of visual cortices in the discrimination between pleasant and unpleasant conditions. The protagonism of this area results expected as the paradigm consists of a visual task. Thus, evoked methodology can extract better properties from the sensorial response than the induced one. However, the induced methodology proves more capable of discriminating conditions in higher order areas where neural oscillation gains a more prominent role in decoding. Taken together, the comparison suggests that both approaches are valid to analyze and extract conclusions from data.

References

[1] Haxby JV. Multivariate pattern analysis of fMRI: The early beginnings. NeuroImage 2012;62:852–5. https://doi.org/10.1016/j.neuroimage.2012.03.016.
[2] Bae G-Y, Luck SJ. Dissociable Decoding of Spatial Attention and Working Memory from EEG Oscillations and Sustained Potentials. J Neurosci 2018;38:409–22. https://doi.org/10.1523/JNEUROSCI.2860-17.2017.
[3] Bae G-Y, Luck SJ. Reactivation of Previous Experiences in a Working Memory Task. Psychol Sci 2019;30:587–95. https://doi.org/10.1177/0956797619830398.
[4] David O, Kilner JM, Friston KJ. Mechanisms of evoked and induced responses in MEG/EEG. Neuroimage 2006;31:1580–91. https://doi.org/10.1016/j.neuroimage.2006.02.034.
[5] Santos-Mayo A, de Echegaray J, Moratti S. Conditioned up and down modulations of short latency gamma band oscillations in visual cortex during fear learning in humans. Sci Rep 2022;12:2652. https://doi.org/10.1038/s41598-022-06596-8.
