# Supplementary material for: Decoding in the Fourth Dimension: Classification of Temporal Patterns and Their Generalization Across Locations
Source: Hum Brain Mapp. 2025 Jan 30;46(2):e70152. doi: 10.1002/hbm.70152 (PMC11780319; doi:10.1002/hbm.70152)
Supplement: Supplementary file 2 — Appendix S2. Supporting Information. [file HBM-46-e70152-s002.pdf]

Supporting Information 2 Appendix

Multi-variate pattern analysis (MVPA), also known as decoding, consists of a multi-variable approach to extract neural representations distributed across neuroimaging recordings. These techniques have been widely in neurosciences fields like brain-computer interface (BCI) research [1], where algorithms focus on reaching the highest decoding rates possibles to detect human brain's activity. However, research in cognitive neuroscience do not emphasize the decoding accuracy of the models, but their capability to explain differences between neural processes. Thus, decoding approaches in cognitive neuroscience has focused on fast and simple classifier models such as supported vector machine (SVM) or linear discriminant analysis (LDA) not because their decoding performance, but its convenience in research [2–4].

Nowadays, there are several decoding approaches and toolboxes [5–8] that allows researchers to analyze electroencephalography recordings (EEG). These tools show two particularities: (1) they conducts their analysis by using space as features for the classifier models, resulting in a time-resolved measurement of the decoders accuracy. (2) Most of these approaches usually employs the SVM algorithm as core for their computation. In contrast, the Time-GAL toolbox proposes a methodology based on using time as features for the classifier model and advocating for the use of the LDA algorithm. Consequently, such differences in decoding design and algorithm may raise concerns about differences in terms of decoding performance.

Here, we address a comparison of both methodologies by replicating the Bae and Luck [4] approach with the same data as used in the article (see figure 3, pleasant vs. unpleasant). This approach is based on the use of SVM algorithm over a 3-folded averaged data. Space (channels or electrodes) is used here as features for the decoding model, thus providing a time-resolve description of the decoding performance. Therefore, this approach employes a procedure

drastically different from the one followed by the time-GAL method. Results for this traditional methodology can be observed in figure S2.1.

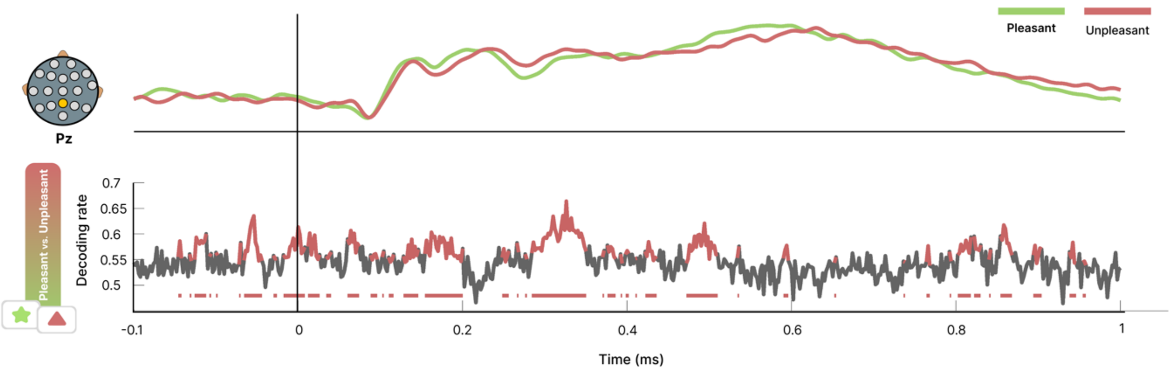

Figure S2.1. Decoding performance for affective pictures following the Bae and Luck [4] procedure. **Top**: Electrophysiological ERP response to the presentation of different emotional content obtained at the parietal central (Pz) electrode for the three cond. **Bottom**: Time-resolved decoding rate for the contrast of pleasant vs. unpleasant pictures. Red sections over the decoding rate as well as red lines below represents time bins where decoding rate is statistically higher than chance-level (uncorrected threshold α = 0.05 for mere visualization). Note that the time-resolved decoding rate line has not been smoothed as done by Bae and Luck [4] in their procedure in order to display exact decoding values.

Particularly, we are interest in the decoding performance of this procedure. The decoding rate is located at 328 ms with a value of 0.664 when using the space as feature for a SVM model, following the Bae and Luck [4] procedure. In contrast, when computing the MVPA analysis using time as features for a LDA model (see Figure 3), the maximum decoding value raises only a value of 0.57 at the electrode 96 (left parietal). These results suggest slightly higher decoding accuracy for the time-resolved procedure using SVM in contrast with the spatial-resolved method based on LDA.

Nevertheless, the procedure conducted here lies not only on the use of SVM models, but also on the average of information. Therefore, a more precise comparison between these results of the spatial decoders and temporal decoders can be obtained focusing on results shown at S1

Appendix. There, we used LDA with averaged information and decoding performance raised a maximal value of 0.592. This decoding maximum value was found also on electrode 96 (right parietal), similarly as done in the manuscript in the single-trial analysis. Based on these results, we could conclude that the use of SVM classifier models and the average of data shows slightly higher decoding capability in terms of pattern recognition accuracy.

Nevertheless, we assume that decoding performance is not the central goal of our procedure. Actually, differences in decoding rate between the three methods (the Time-GAL toolbox, the time-GAL method using averaged information, and Bae and Luck [4] method) are no significant if we compare them to other decoding models obtaining accuracy rates around 0.8 [9,10]. Instead of accuracy, the time-GAL approach is based on the use of temporal information at the single-trial. Therefore, we need to focus on an algorithm capable to rapidly compute pattern classification of neural data to explain differences between conditions.

To measure the suitability of each classifier algorithm in the Time-GAL toolbox, we computed the same data comparison (pleasant vs. unpleasant) varying the model used in the toolbox. Specifically, we selected the LDA, SVM and multi-layer perceptron (MLP) algorithms implemented in MATLAB environment. The two first were selected based on its typical use in neuroimaging decoding [3,5,6]. The MLP classifier was selected to provide a comparison with neural networks. Performance of this perceptron-based implementation is not comparable with deep learning approaches [11], yet the latter requires heavy computational and temporal resources, thereby falling out of the scope of this comparison.

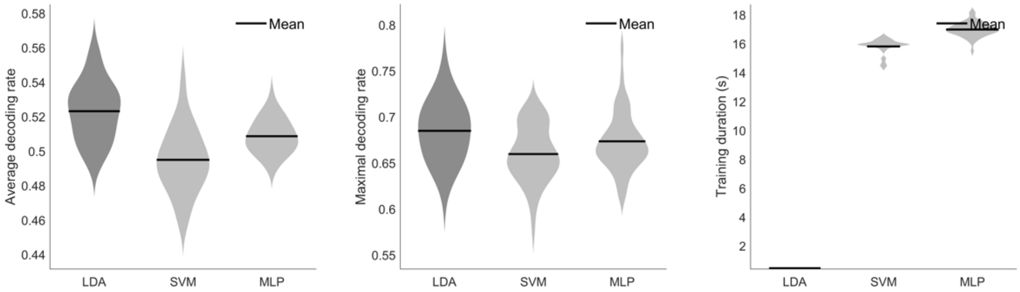

Figure S2.2. Decoding performance and efficiency of linear discriminant analysis (LDA) supported vector machine (SVM) and multi-layer perceptron (MLP) in a leave-one-out procedure using single-trial time as features. **Left**: Average of decoding rate obtained for all channels classifier models per subject. **Middle**: Maximal decoding rate value obtained for all channels classifier models per subject. **Right**: Time elapsed during the training phase for each classifier algorithm.

Results of the comparison are shown in Figure S2.2. They depict the performance and efficiency of the classifier models computing the time-GAL approach on the same data following a leave-one-out validation and using temporal information from single-trials as features for each one of the three models (LDA, SVM, MLP). Violin plots represent the distribution of subjects after averaging the decoding rate (Left), taking the maximal decoding rate (center), and time consumption.

Based on the results, LDA outperforms the other algorithms in terms of both decoding and maximum performance. While this situation was opposite on the case of averaging trials (see above the comparison between Figure 3 and Figure S2.1) where SVM shows better decoding rates, LDA shows a better performance when extracting waveform information from single-trial signals. In the case of the MLP classifier, it does not reach the same average and maximal indices as LDA obtains at the time-GAL analysis. Nevertheless, algorithms do not substantially differ from one to each other in terms of decoding performance, but they significantly do in terms of efficiency. In the case of LDA algorithm, it takes and average of 0.4 s to train the classifier model while this value rises until 15.8 s and 17 s for the SVM and MLP algorithms, respectively.

Taken together, results indicate the suitability of the LDA algorithm to be used as core model for the Time-GAL toolbox as it needs to offer a fast approach to analyze single-trial recordings based on its waveform information. Overall, the fast computation of this model is significant, while the time-GAL approach needs to train a total of channel by subject classifier models. This number dramatically rises as more subjects or channels are included in the analysis, hence efficiency of the classifier model in terms of time becomes critical to offer a suitable toolbox for every researcher regardless of their computational resources.

References

[1] Pawan, Dhiman R. Machine learning techniques for electroencephalogram based brain-computer interface: A systematic literature review. Measurement: Sensors 2023;28:100823. https://doi.org/10.1016/j.measen.2023.100823.

[2] Peelen MV, Downing PE. Testing cognitive theories with multivariate pattern analysis of neuroimaging data. Nat Hum Behav 2023;7:1430–41. https://doi.org/10.1038/s41562-023-01680-z.

[3] Mandelkow H, De Zwart JA, Duyn JH. Linear Discriminant Analysis Achieves High Classification Accuracy for the BOLD fMRI Response to Naturalistic Movie Stimuli. Front Hum Neurosci 2016;10. https://doi.org/10.3389/fnhum.2016.00128.

[4] Bae G-Y, Luck SJ. Dissociable Decoding of Spatial Attention and Working Memory from EEG Oscillations and Sustained Potentials. J Neurosci 2018;38:409–22. https://doi.org/10.1523/JNEUROSCI.2860-17.2017.

[5] Fahrenfort JJ, Van Driel J, Van Gaal S, Olivers CNL. From ERPs to MVPA Using the Amsterdam Decoding and Modeling Toolbox (ADAM). Front Neurosci 2018;12:368. https://doi.org/10.3389/fnins.2018.00368.

[6] López-García D, Peñalver JMG, Górriz JM, Ruz M. MVPAlab: A machine learning decoding toolbox for multidimensional electroencephalography data. Computer Methods and Programs in Biomedicine 2022;214:106549. https://doi.org/10.1016/j.cmpb.2021.106549.

[7] Treder MS. MVPA-Light: A Classification and Regression Toolbox for Multi-Dimensional Data. Front Neurosci 2020;14:289. https://doi.org/10.3389/fnins.2020.00289.

[8] Lu Z, Ku Y. NeuroRA: A Python Toolbox of Representational Analysis From Multi-Modal Neural Data. Front Neuroinform 2020;14:563669. https://doi.org/10.3389/fninf.2020.563669.

[9] Parto Dezfouli M, Daliri MR. Single-Trial Decoding from Local Field Potential Using Bag of Word Representation. Brain Topogr 2020;33:10–21. https://doi.org/10.1007/s10548-019-00726-8.

[10] Daliri MR. A hybrid method for the decoding of spatial attention using the MEG brain signals. Biomedical Signal Processing and Control 2014;10:308–12. https://doi.org/10.1016/j.bspc.2012.12.005.

[11] Wang X, Liang X, Jiang Z, Nguchu BA, Zhou Y, Wang Y, et al. Decoding and mapping task states of the human brain via deep learning. Human Brain Mapping 2020;41:1505–19. https://doi.org/10.1002/hbm.24891.
